# Gender disparities in patients undergoing extracorporeal cardiopulmonary resuscitation

**DOI:** 10.3389/fcvm.2023.1265978

**Published:** 2024-01-16

**Authors:** A. Springer, A. Dreher, J. Reimers, L. Kaiser, E. Bahlmann, H. van der Schalk, P. Wohlmuth, N. Gessler, K. Hassan, J. Wietz, B. Bein, T. Spangenberg, S. Willems, S. Hakmi, E. Tigges

**Affiliations:** ^1^ Department of Cardiology and Critical Care, Asklepios Clinic St. Georg, Hamburg, Germany; ^2^ Asklepios ProResearch, Hamburg, Germany; ^3^ DZHK (German Centre for Cardiovascular Research), Partner Site Hamburg/Kiel/Luebeck, Hamburg, Germany; ^4^ Department of Cardiac Surgery, Asklepios Clinic St. Georg, Hamburg, Germany; ^5^ Department of Emergency Medicine, Asklepios Clinic St. Georg, Hamburg, Germany; ^6^ Department of Anaesthesiology and Critical Care, Asklepios Clinic St. Georg, Hamburg, Germany; ^7^ Department of Cardiology and Critical Care, Asklepios Clinic Altona, Hamburg, Germany; ^8^ Semmelweis-University, Budapest, Hungary

**Keywords:** eCPR, VA-ECMO, OHCA, gender, CPR

## Abstract

**Introduction:**

The use of venoarterial extracorporeal membrane oxygenation (VA-ECMO) in extracorporeal cardiopulmonary resuscitation (eCPR) has emerged as a treatment option for selected patients who are experiencing refractory cardiac arrest (CA). In the light of increasing availability, the analyses of outcome-relevant predisposing characteristics are of growing importance. We evaluated the prognostic influence of gender in patients presenting with out-of-hospital cardiac arrest (OHCA) treated with eCPR.

**Methods:**

We retrospectively analysed the data of 377 consecutive patients treated for OHCA using eCPR in our cardiac arrest centre from January 2016 to December 2022. The primary outcome was defined as the survival of patients until they were discharged from the hospital, with a favourable neurological outcome [cerebral performance category (CPC) score of ≤2]. Statistical analyses were performed using baseline comparison, survival analysis, and multivariable analyses.

**Results:**

Out of the 377 patients included in the study, 69 (21%) were female. Female patients showed a lower prevalence rate of pre-existing coronary artery disease (48% vs. 75%, *p* < 0.001) and cardiomyopathy (17% vs. 34%, *p* = 0.01) compared with the male patients, while the mean age and prevalence rate of other cardiovascular risk factors were balanced. The primary reason for CA differed significantly (female: coronary event 45%, pulmonary embolism 23%, cardiogenic shock 17%; male: coronary event 70%, primary arrhythmia 10%, cardiogenic shock 10%; *p* = 0.001). The prevalence rate of witnessed collapse (97% vs. 86%; *p* = 0.016) and performance of bystander CPR (94% vs. 85%; *p* = 0.065) was higher in female patients. The mean time from collapse to the initiation of eCPR did not differ between the two groups (77 ± 39 min vs. 80 ± 37 min; *p* = 0.61). Overall, female patients showed a higher percentage of neurologically favourable survival (23% vs. 12%; *p* = 0.027) despite a higher prevalence of procedure-associated bleeding complications (33% vs. 16%, *p* = 0.002). The multivariable analysis identified a shorter total CPR duration (*p* = 0.001) and performance of bystander CPR (*p* = 0.03) to be associated with superior neurological outcomes. The bivariate analysis showed relevant interactions between gender and body mass index (BMI).

**Conclusion:**

Our analysis suggests a significant survival benefit for female patients who obtain eCPR, possibly driven by a higher prevalence of witnessed collapse and bystander CPR. Interestingly, the impact of patient age and BMI on neurologically favourable outcome was higher in female patients than in male patients, warranting further investigation.

## 1 Introduction

Out-of-hospital cardiac arrest (OHCA) is one of the leading causes of death in Europe, with an incidence rate ranging from 67 to 170 per 100,000 person-years and persistently low rates of survival (1). Recently, extracorporeal cardiopulmonary resuscitation (eCPR) has emerged as a treatment option for refractory cardiac arrest (CA). Albeit recently published randomised controlled trials have displayed variable results, the number of cases and medical centres adopting eCPR therapy has increased rapidly, possibly driven by reports of favourable outcomes in retrospective analyses and the positive results of the ARREST trial (2–5). However, patient selection seems to be of incremental importance, given the highly invasive nature and the consecutive risk for complications, as well as the considerably high demand in personnel and resources of this treatment option (6).

Although certain factors, such as witnessed cardiac arrest, short overall resuscitation duration, and refractory ventricular fibrillation, have been identified as having a positive effect on outcome measures, the impact of gender on these measures remains unclear. Two-thirds of cardiac arrest patients are male. Nonetheless, female patients remain underrepresented in the current randomised controlled trials, with only 17% of the participants being of female gender in the ARREST and Prague OHCA studies (2, 3). Studies have suggested that women exhibit overall lower survival rates and worse neurological outcomes after conventional CPR for CA compared with men (7–9). When analysing women of reproductive age, these effects seem to be suspended, with favourable outcomes reported in this collective (10). Whether these gender differences exist in the context of eCPR remains widely unknown, with only limited data available reporting inconsistent results (11, 12). We aimed to assess the impact of gender on outcomes in a diverse group of patients treated with eCPR for OHCA at our cardiac arrest centre.

## 2 Methods

### 2.1 Patient selection

We present retrospective data obtained from a single-centre registry of patients treated with eCPR for refractory OHCA at the Cardiac Arrest Center (CAC) of the Asklepios Clinic St. Georg (Hamburg, Germany). During the period of the analysis, spanning from January 2016 to December 2022, a total of 377 patients were treated with VA-ECMO at our centre. Excluding 47 patients with a significant amount of missing data, the study focused on 330 cases of patients treated with eCPR for OHCA. The primary outcome was defined as the survival of patients until they were discharged from the hospital with a favourable neurological outcome [cerebral performance category (CPC) score of ≤2]. The secondary outcome was defined as the survival of patients until they were discharged from the hospital, regardless of the CPC scoring. The study protocol was approved by the local ethics committee (2023-300393-WF).

### 2.2 Statistical analyses

All cases were categorised into gender-specific subgroups depending on their biological gender. The two groups were characterised, and baseline characteristics were compared using Fisher’s exact test, Pearson's chi-squared test, and Wilcoxon rank sum test when applicable. Continuous data were summarised as means ± standard deviations or as medians (25th and 75th percentiles) as appropriate. The categorical data were presented as *N* (%). Overall survival and survival with good neurological outcome (CPC ≤ 2) were visualised using Kaplan–Meier plots. Regarding the survival endpoint with good neurological outcome, patients who were discharged alive but had demonstrated poor neurological status were assigned the discharge time as the event time.

The variables considered in the Cox proportional hazards model for determining survival with good neurological outcome were age, body mass index (BMI), witnessed collapse (yes/no), bystander cardiopulmonary resuscitation (yes/no), initial ECG (VF or VT), the administration of sodium bicarbonate (yes/no), amiodarone, and epinephrine, and the duration from collapse to the initiation of eCPR. The interaction effects between each variable and gender were taken into account. Continuous variables (age, duration to eCPR, BMI) were transformed in advance using restricted cubic spline functions with three knots to consider non-linearity. All model terms were shown with parameter estimates, standard errors, Wald statistics and *p*-values. The effects on mortality were presented as hazard ratios and 95% confidence intervals. The interactions between gender and BMI and between gender and age were shown using log relative hazard plots and figures presenting model-based survival predictions. All *p*-values were two-sided, and a *p*-value < 0.05 was considered significant. All calculations were performed using the statistical analysis software R (R Core Team, 2023).

### 2.3 eCPR program

Embedded in a tertiary care hospital in the urban area of Hamburg (Germany), our CAC falls back on a long-term experience with VA-ECMO implantation and management with a focus on eCPR. We offer an around-the-clock ECMO service with a specialised intensive care unit, as well as specialised heart failure and chest pain units.

In case of OHCA patients arriving to the emergency department, the interdisciplinary cardiac arrest receiving team (CART) is alerted beforehand. The CART comprises personnel from the departments of interventional cardiology, anaesthesiology, and emergency medicine. Based on the presumed aetiology, other departments are alerted simultaneously. Treatment decision is made upon arrival at a “cardiac arrest fast assessment area” based on the current expert opinion as well as individual criteria with a focus on the avoidance of time delay. Positive indicators for eCPR initiation are witnessed collapse, performance of bystander CPR, no-flow-time of <5 min, low-flow-time of <60 min, age <75 years (y), and shockable initial rhythm. However, the final decision is left to the CART, and no strict criteria for deferral are provided. VA-ECMO implantation is performed in the cardiac catheter laboratory under fluoroscopic guidance. In the absence of contraindications, uni- or bilateral peripheral femoral access is used, and cannulation is performed via Seldinger's technique. To prevent peripheral limb ischaemia, the implantation of a distal perfusion cannula when feasible is the standard practice. Postinterventional further diagnostics include a coronary angiography and an individualised computed tomography. Intensive care management is at the discretion of the intensive care specialist, following the current guidelines for treatment of the underlying medical condition (e.g., cardiogenic shock, acute myocardial infarction, pulmonary embolism), as well as post-resuscitation care (13–15). Cerebral performance category scoring is performed routinely by trained personnel at the time of discharge from the intensive care unit or prior to transfer to a neurological rehabilitation unit.

## 3 Results

### 3.1 Patient characteristics

The studied collective was predominantly male (female gender *n* = 69, 21%). No relevant difference in age was found. Male patients were taller and heavier than female patients, without exhibiting differences in body mass index. Male patients showed a higher prevalence of pre-existing coronary artery disease (CAD) and consecutively higher rates of ischaemic cardiomyopathy (ICM), as well as prior operative myocardial revascularisation. The rates of prior percutaneous coronary interventions (PCI) were higher in male patients as well, although without reaching statistical significance. Female patients on the other hand were seen to have a higher prevalence of prior pulmonary embolism. Prevalence of known cardiovascular risk factors and comorbidities were balanced (Table 1).

**Table 1:** Patient characteristics.

	All *n* = 330	Male *n* = 261	Female *n* = 69	*p*-value^a^
Age (year)				0.52
Mean (SD)	59 (13)	59 (13)	57 (15)	
Height (m)				<0.001
Mean (SD)	1.75 (0.07)	1.78 (0.06)	1.67 (0.06)	
Weight (kg)				0.001
Mean (SD)	85 (18)	87 (16)	79 (20)	
BMI (kg/m^2^)				0.29
Mean (SD)	27.7 (5.4)	27.6 (4.9)	28.4 (7.0)	
Coronary artery disease, % (*n*)	69 (223)	75 (191)	48 (32)	<0.001
Peripheral artery disease, % (*n*)	12 (32)	13 (28)	6.3 (4)	0.14
Chronic kidney disease, % (*n*)	16 (44)	16 (34)	15 (10)	0.92
Any cardiomyopathy, % (*n*)	30 (85)	34 (74)	17 (11)	0.010
Dilated cardiomyopathy, % (*n*)	4.0 (11)	3.3 (7)	6.3 (4)	0.28
Ischaemic cardiomyopathy, % (*n*)	21 (58)	26 (56)	3.2 (2)	<0.001
Other cardiomyopathy, % (*n*)	3.6 (10)	3.3 (7)	4.8 (3)	0.70
Chronic pulmonary disease, % (*n*)	6.1 (16)	6.4 (13)	4.8 (3)	0.77
Atrial fibrillation, % (*n*)	23 (62)	23 (47)	25 (15)	0.80
Hypertension, % (*n*)	55 (133)	56 (101)	52 (32)	0.65
Diabetes (Type 1 and 2), % (*n*)	30 (75)	29 (56)	32 (19)	0.69
Hereditary predisposition for cardiovascular events, % (*n*)	7.7 (17)	7.2 (12)	9.1 (5)	0.77
Hyperlipoproteinaemia, % (*n*)	28 (67)	28 (50)	29 (17)	0.86
Nicotine abuse, % (*n*)	33 (74)	36 (60)	25 (14)	0.13
Prior pulmonary embolism, % (*n*)	1.2 (3)	0 (0)	5.0 (3)	0.013
Prior PCI, % (*n*)	19 (58)	21 (51)	11 (7)	0.054
Implanted pacemaker, % (*n*)	3.9 (12)	4.5 (11)	1.5 (1)	0.47
Implanted cardioverter defibrillator, % (*n*)	2.9 (9)	3.2 (8)	1.5 (1)	0.69
Implanted cardiac resynchronisation pacemaker, % (*n*)	0.3 (1)	0.4 (1)	0 (0)	>0.99
Implanted cardiac resynchronisation defibrillator, % (*n*)	1.9 (6)	2.4 (6)	0 (0)	0.35
Prior CABG, % (*n*)	11 (35)	13 (33)	3.0 (2)	0.017
Prior non-coronary cardiac surgery, % (*n*)	3.2 (10)	2.9 (7)	4.5 (3)	0.46
Prior MitraClip intervention, % (*n*)	2.9 (9)	2.5 (6)	4.5 (3)	0.41
Prior TAVR, % (*n*)	2.9 (9)	2.4 (6)	4.5 (3)	0.41

^a^ Wilcoxon rank sum test; Pearson's chi-squared test; Fisher's exact test.

### 3.2 Prehospital data

The overall prevalence of witnessed collapse and bystander CPR was high. Initial rhythm was shockable in 50% of the cases. Female patients had consistently higher rates of witnessed collapse and showed a tendency for a higher prevalence of bystander CPR. A shockable rhythm was observed more frequently in male patients, while no overall statistically significant difference was noted. Furthermore, female patients had a shorter time to initiation of advanced life support (ALS) CPR, e.g., the arrival of trained emergency medicine service (EMS) teams. Overall, the median resuscitation duration from collapse to the initiation of eCPR was 75 minutes (min) and did not differ between the gender-specific subgroups (Table 2).

**Table 2:** Prehospital data.

	All *n* = 330	Male *n* = 261	Female *n* = 69	*p*-value^a^
Witnessed collapse, % (*n*)	88 (276)	86 (213)	97 (63)	0.016
Bystander CPR, % (*n*)	87 (279)	85 (217)	94 (62)	0.065
Initial ECG, % (*n*)				0.22
VT/VF	50 (152)	52 (125)	44 (27)	
PEA/asystole	50 (149)	48 (114)	56 (35)	
Mechanical CPR device usage, % (*n*)	84 (147)	86 (127)	74 (20)	0.10
Time from collapse to ALS CPR (min)				0.045
Median [Q1, Q3]	4.0 [0.0, 9.5]	5.0 [0.0, 10.0]	0.0 [0.0, 6.7]	
Time from collapse to ECPR (min)				0.61
Median [Q1, Q3]	75 [58, 95]	75 [56, 95]	75 [64, 92]	

^a^ Pearson's chi-squared test; Wilcoxon rank sum test.

### 3.3 Procedure data and complications

While the primary reason for cardiac arrest in the entire collective and both subgroups was an acute coronary event, this was more pronounced in male patients, with 70% of the cases presenting this presumed aetiology. The prevalence of the acute coronary event in female patients was lower (45%), while the rates of pulmonary embolism where considerably higher in female patients than that in male patients. Other relevant presumed origins of CA were cardiogenic shock and primarily arrhythmogenic. The median door-to-ECMO time was 15 min, and no gender-specific differences were apparent. ECMO implantation was followed-up with primary implantation of an active left ventricular (LV) unloading device in 15% of the cases. Overall, the median ECMO runtime was 1 day and comparable in both subgroups (Table 3).

**Table 3:** Procedure data.

	All *n* = 330	Male *n* = 261	Female *n* = 69	*p*-value^a^
Door-to-ECMO time (min)				0.88
Median [Q1, Q3]	15 [13, 25]	15 [15, 25]	17 [10, 25]	
Left ventricular venting device, % (*n*)				0.43
Impella 2.5	7.7 (25)	8.5 (22)	4.5 (3)	
Impella CP	7.7 (25)	8.1 (21)	6.0 (4)	
None	84.6 (275)	83.4 (215)	89.5 (60)	
Diagnosis after procedure, % (*n*)				0.001
Coronary event	65 (161)	70 (140)	45 (21)	
Primarily arrhythmogenic	9.3 (23)	10.0 (20)	6.4 (3)	
Non-ischaemic cardiogenic shock	11 (28)	10.0 (20)	17 (8)	
Takotsubo cardiomyopathy	1.2 (3)	1.0 (2)	2.1 (1)	
Aortic dissection	3.2 (8)	2.5 (5)	6.4 (3)	
Pulmonary embolism	8.9 (22)	5.5 (11)	23 (11)	
Hypothermia	1.2 (3)	1.5 (3)	0 (0)	
Unknown	82	60	22	
Total ECMO runtime (days)				0.27
Median [Q1, Q3]	1.00 [0.00, 3.00]	1.00 [1.00, 3.00]	1.00 [0.00, 3.00]	

^a^ Wilcoxon rank sum test; Pearson's chi-squared test; Fisher's exact test.

Female patients displayed higher rates of periprocedural bleeding complications compared with male patients, with consequently higher need for interventional and/or surgical measures to treat these adverse events. No difference was seen in other relevant periprocedural complications (Table 4).

**Table 4:** Complications.

** **	All *n* = 330	Male *n* = 261	Female *n* = 69	*p*-value^a^
Visceral laceration, % (*n*)	2.8 (9)	2.7 (7)	2.9 (2)	>0.99
Hemothorax, % (*n*)	4.9 (16)	5.5 (14)	2.9 (2)	0.54
Relevant bleeding, % (*n*)	20 (65)	16 (42)	33 (23)	0.002
Complications requiring surgical or interventional measures, % (*n*)	12 (39)	10 (26)	19 (13)	0.046

^a^ Wilcoxon rank sum test; Pearson's chi-squared test; Fisher's exact test.

### 3.4 Outcome

**Figure 1:**
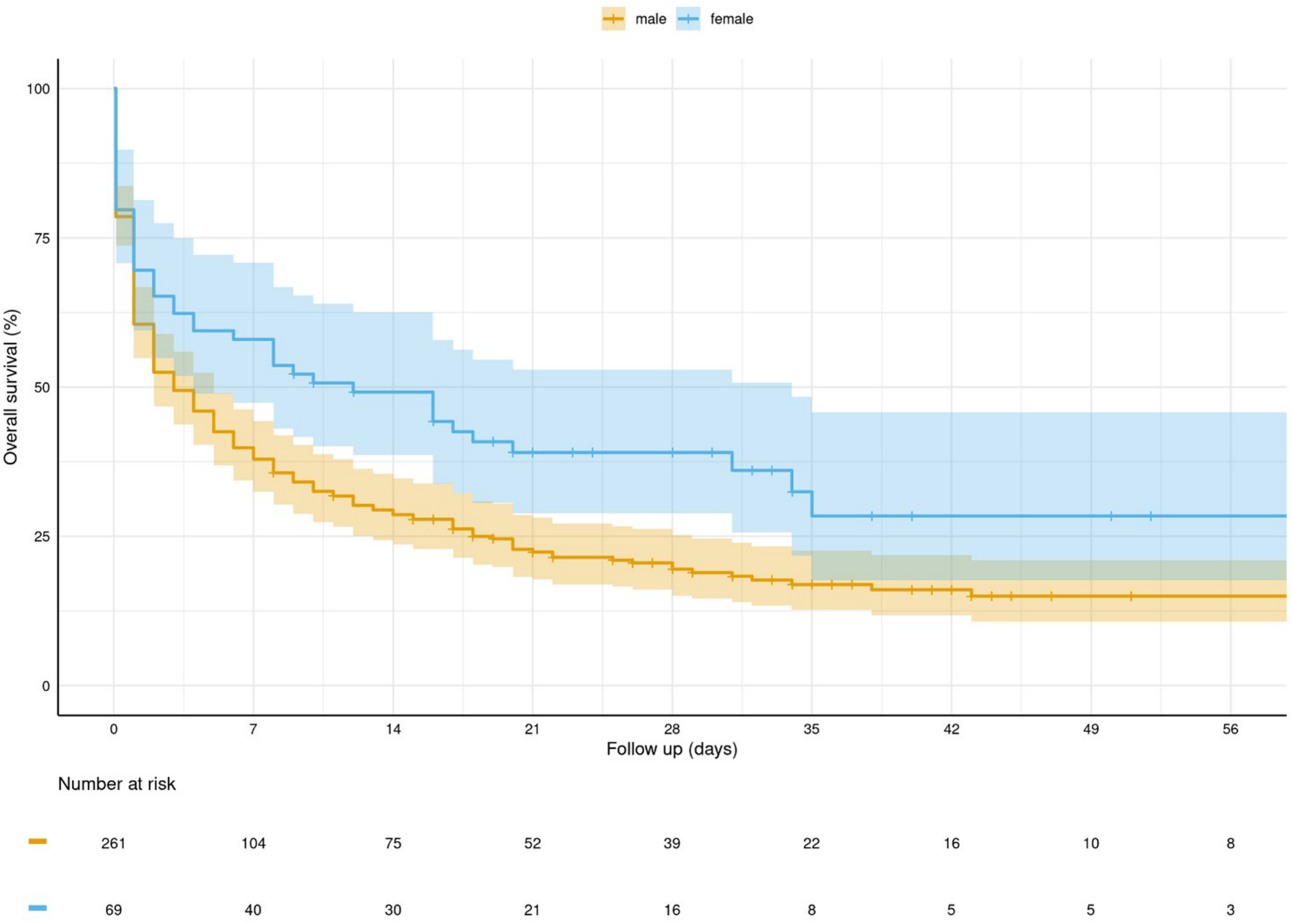
Kaplan–Meier curve for survival stratified by gender.

**Figure 2:**
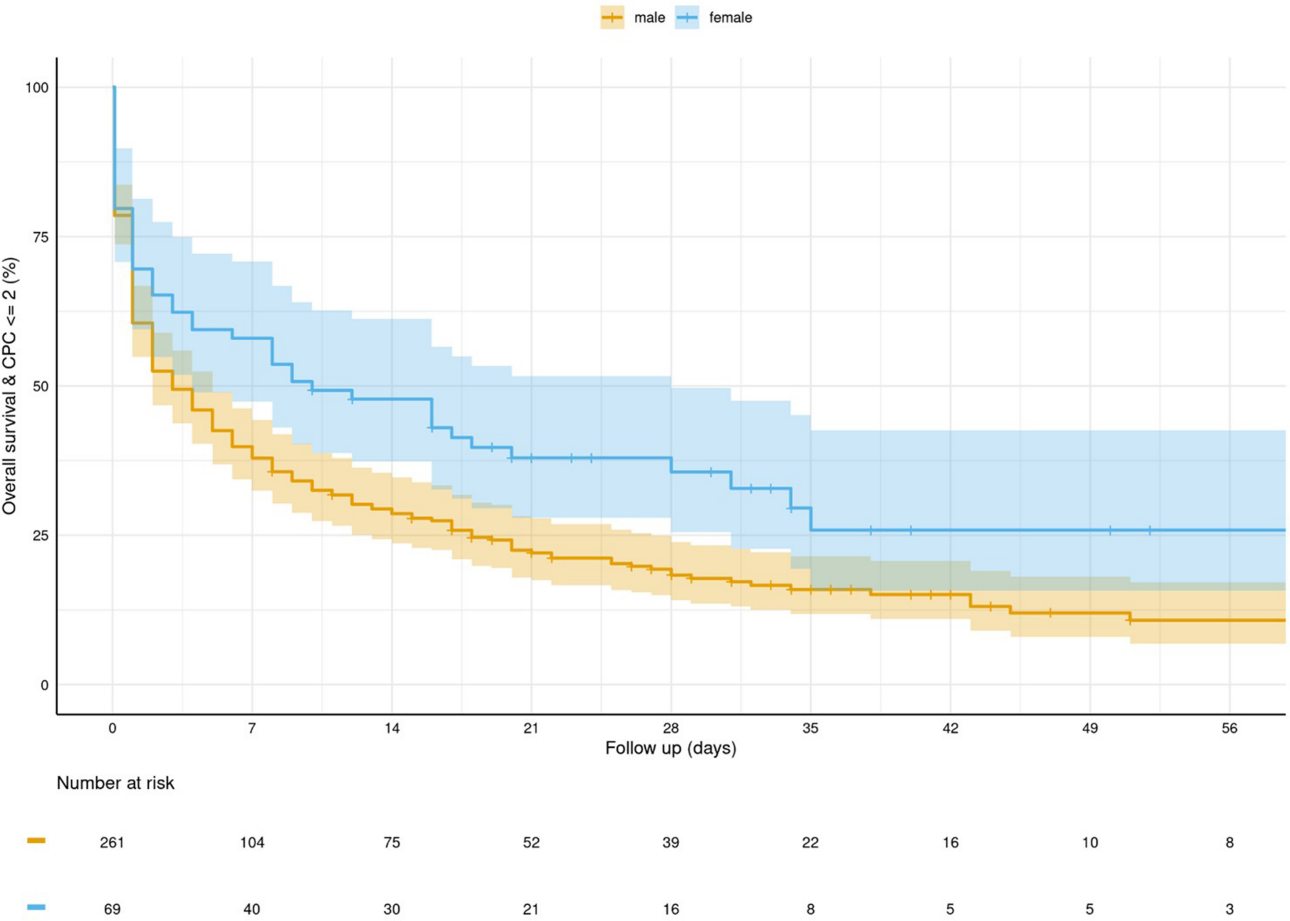
Kaplan–Meier curve for survival and favourable neurological outcome (CPC ≤ 2) stratified by gender.

Out of the 330 studied patients, 70 survived up to hospital discharge (21%), and 44 (14%) patients showed favourable neurological outcomes, defined as a CPC score of ≤2. Female patients had significantly higher rates of survival (Figure 1), as well as higher rates of neurologically favourable outcomes at the time of discharge (Table 5, Figure 2).

**Table 5:** Outcome.

	All *n* = 330	Male *n* = 261	Female *n* = 69	*p*-value^a^
Survival up to hospital discharge, % (*n*)	21 (70)	18 (46)	35 (24)	0.002
Discharge CPC ≤ 2, % (*n*)	14 (44)	12 (30)	23 (14)	0.027

^a^ Pearson's chi-squared test.

### 3.5 Multivariable survival model

The aggravating influence on adverse outcome was highest for an initially non-shockable rhythm and a higher BMI. Unwitnessed collapse, no performance of bystander CPR, and a longer time from collapse to eCPR initiation showed a tendency for being associated with worse neurological outcomes, without reaching statistical significance. Patients, in whom no epinephrine was administered, were more likely to survive with favourable neurological outcomes (Table 6).

**Table 6:** Hazard ratio by multivariable analysis.

	Hazard ratio	CI low	CI high
Higher age (Q3: 68 vs. Q1: 51 years)	1.012	0.849	1.206
Higher BMI (Q3: 29.5 vs. Q1: 24.5 kg/m^2^)	1.244	1.019	1.518
No witnessed collapse	1.143	0.975	1.340
No bystander CPR	1.533	0.995	2.363
Non-shockable initial rhythm	1.447	1.066	1.966
No administration of epinephrine	0.598	0.394	0.907
Administration of amiodarone	0.763	0.553	1.052
Administration of sodium bicarbonate	0.803	0.574	1.124
Longer time to ECPR (Q3: 91 vs. Q1: 57 min)	1.143	0.975	1.340

Hazard ratios and 95% confidence intervals derived from a Cox proportional hazards model predicting survival with good neurologic outcome from baseline data and their interaction with gender (model summary shown in Table 7). Values are adjusted to male gender, median age, median BMI, median time to eCPR, witnessed collapse, bystander CPR, epinephrine, no administration of amiodarone, and no administration of sodium bicarbonate.

The multivariable analysis revealed time from collapse to eCPR initiation, non-shockable initial rhythm, and administration of epinephrine to be independently associated with outcome, while the effect of age and BMI seemed to be gender dependent (Tables 7, 8).

**Table 7:** Main effect term.

	Parameter estimate	Std. error	Wald statistics	*p*-value
Female gender	1.221	2.498	0.489	0.625
Age (linear)	−0.016	0.012	−1.384	0.166
Age (non-linear)	0.021	0.014	1.527	0.127
BMI (linear)	0.062	0.042	1.490	0.136
BMI (non-linear)	−0.046	0.064	−0.715	0.475
Witnessed collapse	−0.085	0.224	−0.381	0.703
Bystander CPR	−0.427	0.221	−1.937	0.053
Non-shockable rhythm (PEA/asystole)	0.370	0.156	2.366	0.018
Administration of epinephrine	0.514	0.212	2.419	0.016
Administration of amiodarone	−0.271	0.164	−1.649	0.099
Administration of sodium bicarbonate	−0.219	0.171	−1.278	0.201
Time from collapse to ECPR (linear)	0.016	0.005	3.307	0.001
Time from collapse to ECPR (non-linear)	−0.014	0.004	−3.587	0.000

Summary of Cox proportional hazards model predicting survival with good neurologic outcome from baseline data and their interaction with gender. Continuous data were non-linearly transformed. Parameter estimates, standard errors, Wald statistics, and *p*-values are shown.

**Table 8:** Interacting term (corrected by gender).

** **	Parameter estimate	Std. error	Wald statistics	*p*-value
Age (linear)	0.081	0.028	2.929	0.003
Age (non-linear)	−0.090	0.036	−2.471	0.013
BMI (linear)	−0.220	0.069	−3.197	0.001
BMI (non-linear)	0.301	0.107	2.818	0.005
Witnessed collapse	0.946	1,172	0.807	0.420
Bystander CPR	−0.202	0.635	−0.318	0.751
Non-shockable rhythm (PEA/asystole)	−0.797	0.442	−1.802	0.072
Administration of epinephrine	−0.069	0.447	−0.155	0.877
Administration of amiodarone	−0.994	0.508	−1.957	0.050
Administration of sodium bicarbonate	−0.097	0.493	−0.198	0.843
Time from collapse to ECPR (linear)	−0.007	0.011	−0.667	0.505
Time from collapse to ECPR (non-linear)	0.011	0.010	1.181	0.237

Summary of Cox proportional hazards model predicting survival with good neurologic outcome from baseline data and their interaction with gender. Continuous data were non-linearly transformed. Parameter estimates, standard errors, Wald statistics, and *p*-values are shown.

### 3.6 Relevant interactions

**Figure 3:**
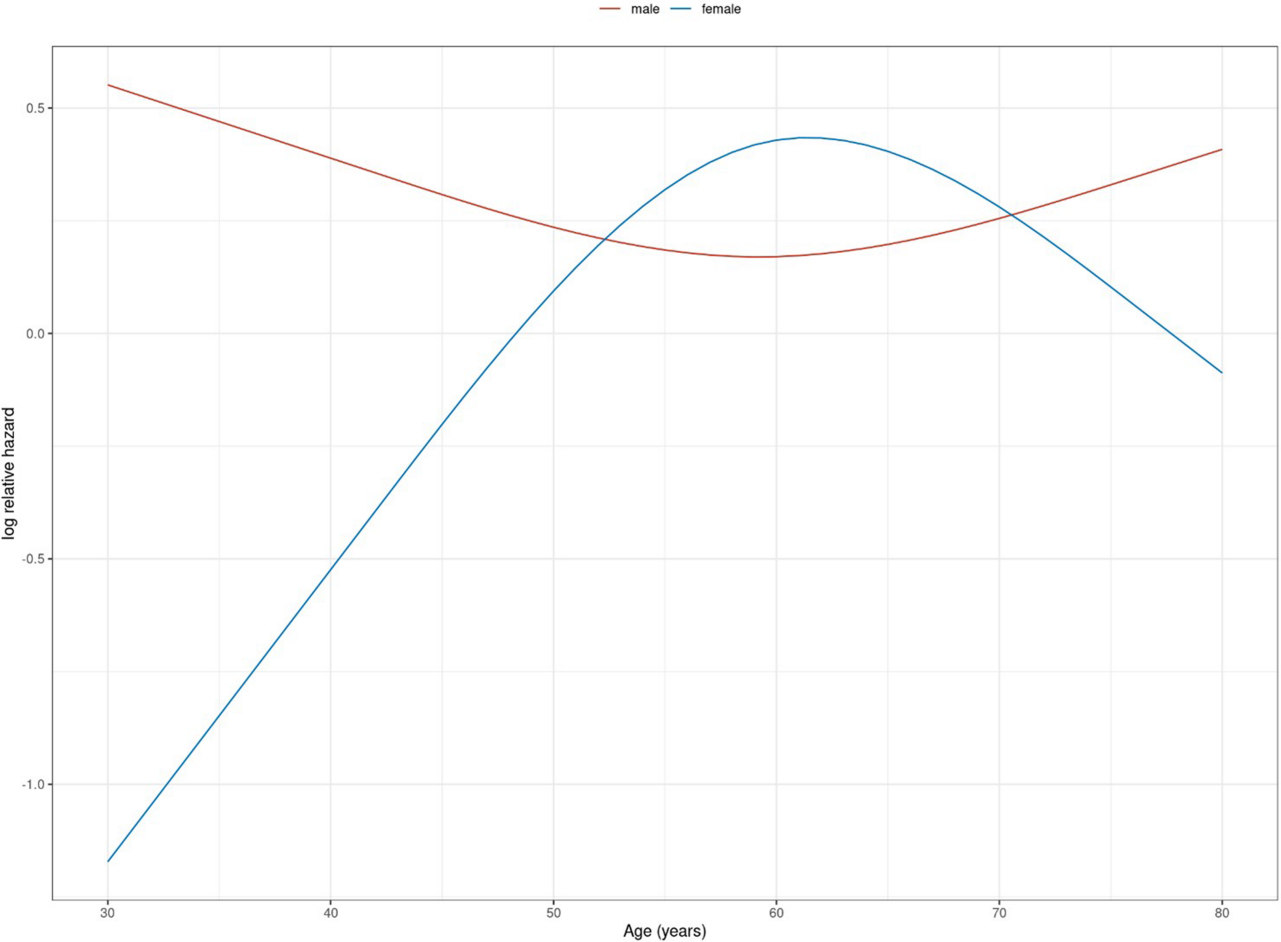
Logarithmic relative hazard for age (in years) on adverse neurological outcome (death or survival with CPC > 2) stratified by gender.

**Figure 4:**
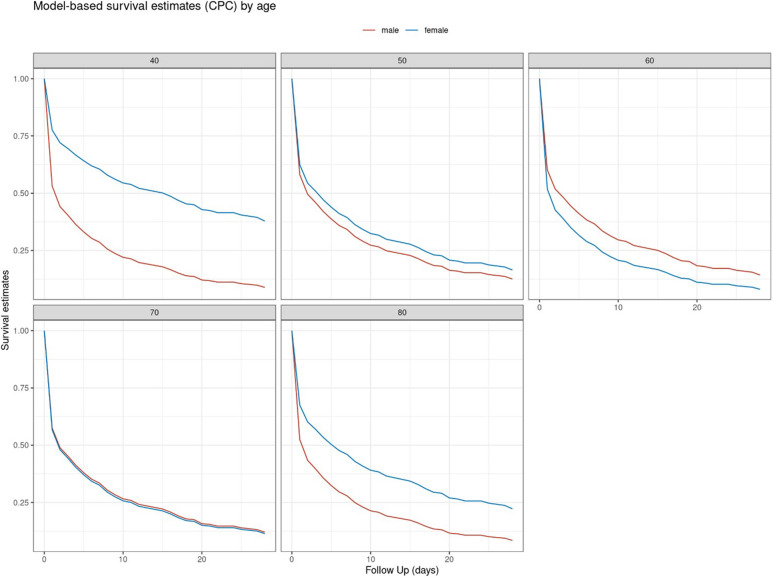
Model-based based estimate for neurologically favourable survival rates (CPC ≤ 2) for selected age values (in years) stratified by gender.

Relevant interactions between gender and suspected risk factor for an adverse neurological outcome were observed in the case of age and BMI. Female patients exhibited an almost linear increase of risk for adverse neurological outcome with increasing age up to an age of 60 years. In male patients the aforementioned risk was higher at a younger age and decreased slightly with increasing age. Above 60 years of age, the adverse impact of age decreased in female patients and slightly increased in male patients (Figure 3). Hence, the model-based estimates for neurologically favourable survival rates according to age differed between female and male patients (Figure 4).

**Figure 5:**
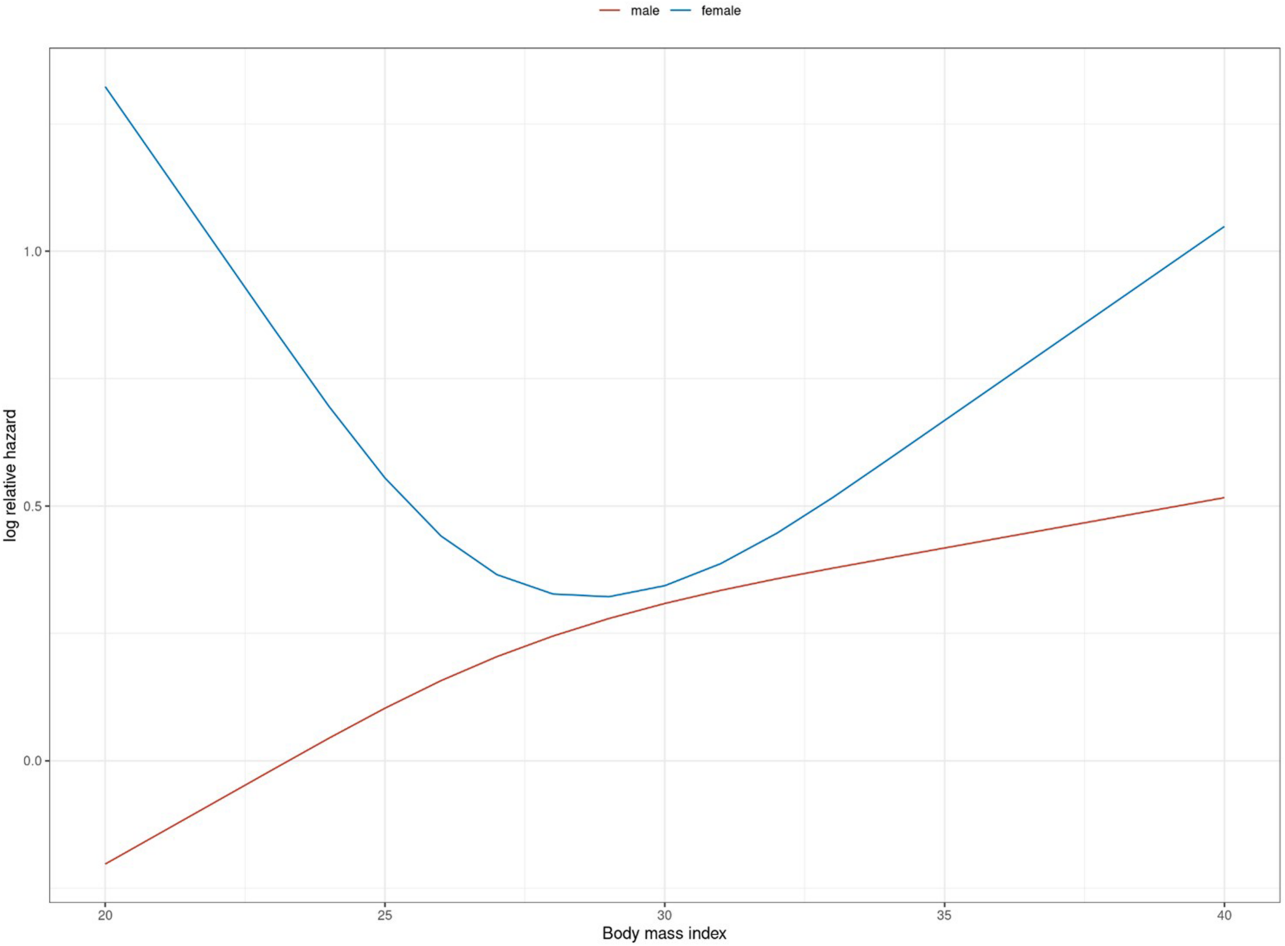
Logarithmic relative hazard for BMI (in kg/m^2^) on adverse neurological outcome (death or survival with CPC > 2) stratified by gender.

**Figure 6:**
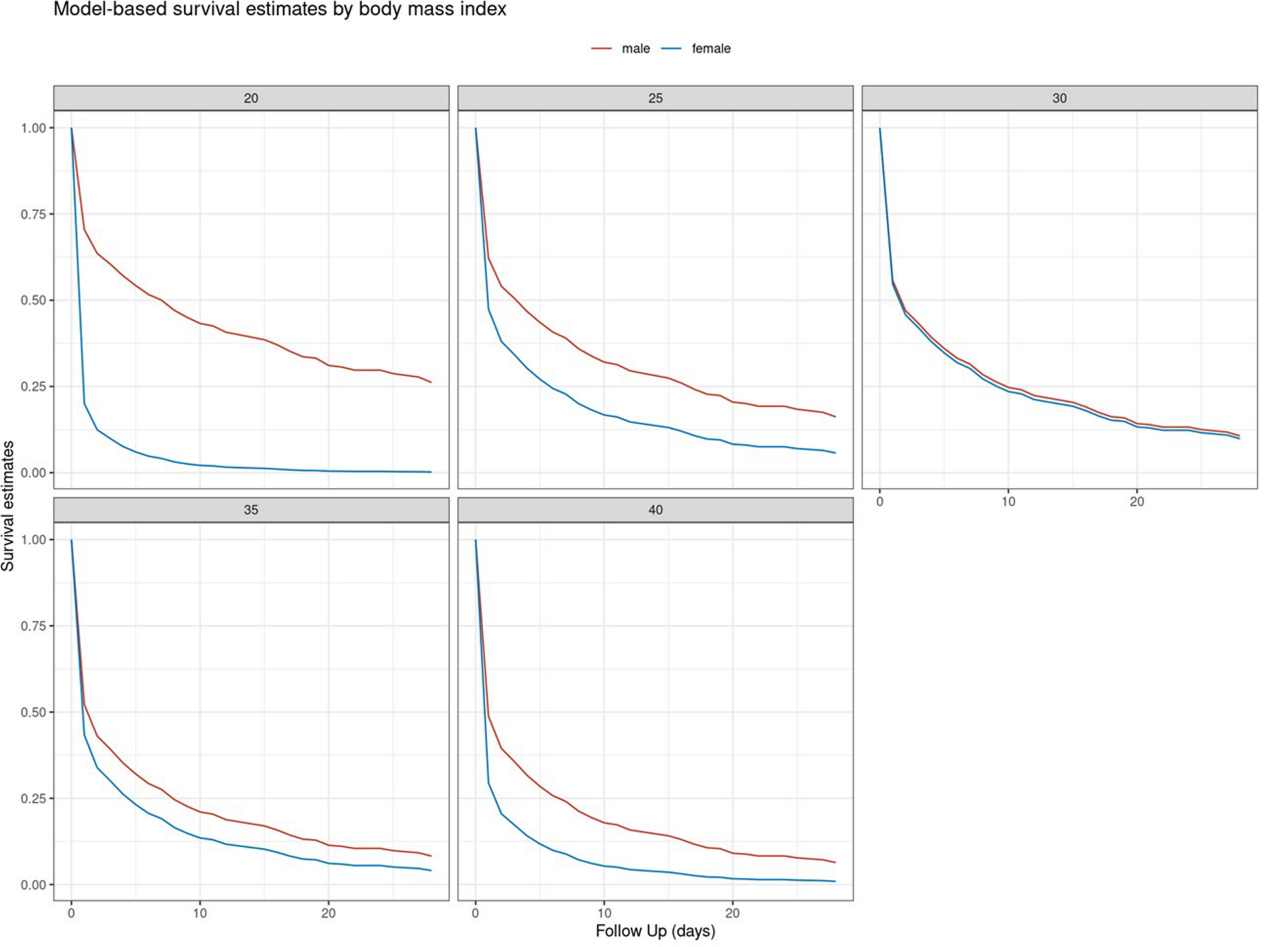
Model-based based estimate for neurologically favourable survival rates (CPC ≤ 2) for selected BMI values (in kg/m^2^) stratified by gender.

In male patients, the increasing BMI values were associated with an almost linear increase of risk for death or neurologically unfavourable outcomes. In female patients on the other hand, low and high BMI values were equally associated with a higher risk for adverse outcomes, with relevant relative risk reduction in the range of 25–33 kg/m^2^. Overall, female patients seemed to suffer a higher impact of BMI on adverse outcome than male patients did (Figure 5). The model-based estimates for neurologically favourable survival rates by BMI differed accordingly (Figure 6).

## 4 Discussion

In our study of a large all-comers collective of patients undergoing eCPR for OHCA, female patients had significantly higher rates of overall, as well as neurologically favourable, survival. Gender-independent survival was 21%; 14% of patients were discharged with favourable neurological outcomes. The suspected aetiology of cardiac arrest differed between the gender-specific subgroups, with female patients presenting lower rates of acute coronary events than male patients and exhibiting more cases of pulmonary embolism. Bleeding complications were observed more frequently in female patients.

The multivariable analysis revealed that the time from collapse to eCPR was independently associated with outcome, and the effect of age and BMI was ascertained to be gender dependent. A significant influence on adverse outcome was observed for higher age, higher BMI, and non-shockable initial rhythm.

The stated survival rates and percentage of patients discharged with a favourable neurological outcome is consistent with the current literature (4, 16), although the data should be evaluated under the constraint that we describe an all-comers collective recorded over a long time interval with an inherent learning curve. The discrepancy to the higher survival rates in current RCTs (2, 3) could arguably be due to the lower rates of shockable primary rhythm and longer time from collapse to eCPR initiation in our collective. A possible explanation of the longer overall resuscitation duration seen in our collective might be the limited availability of mechanical CPR (mCPR) devices in the studied region until December 2020. Prior to this date, these devices had to be requested from the EMS provided by the German army and separately transported to the resuscitation location. This might account for a possible time delay in transport and consecutively longer resuscitation duration until eCPR was established.

We hypothesise that the driving factors in outcome superiority in female patients were the significantly higher rates of witnessed collapse, tendency to more frequent performance of bystander CPR, and overall shorter time to initiation of ALS CPR. Opposing our results, the recent meta-analyses of CA collectives without focusing on eCPR do not support a survival benefit for female patients. This could be due to overall lower rates of witnessed collapse and bystander CPR and higher rates of non-shockable rhythm compared with our collective (9, 17), although the overall comparability is impaired due to the selected eCPR cohort we describe. Interestingly, small sample studies on patients undergoing eCPR for OHCA with similar baseline characteristics as the present analysis support our results suggesting favourable outcomes for female patients, particularly of younger age, speculating that higher blood levels of oestrogen might have a beneficial impact on cardiac regeneration and inflammation after CA (11, 18, 19).

Hormonal influences on beneficial outcome could explain the apparent gender dependence of age in our studied collective, with female patients of reproductive age displaying increased rates of neurologically favourable outcome compared with male patients of the same age category.

Large-scale data regarding gender differences in outcome in the context of eCPR, as well as in-depth analyses of possible hormonal effects, remain to be provided.

In our collective, female patients showed significantly lower rates of pre-existing CAD and ICM, which was also reflected in a lower percentage of acute coronary events as the suspected cause of cardiac arrest. Taking into account the higher rates of pulmonary embolism in female gender, this finding suggests that there might be considerable gender-specific differences in the events causing OHCA, calling for an individualised approach. These findings are consistent with the data from recent meta-analyses stating lower rates of acute coronary events as CA aetiology in female patients (9, 17), as well as observational studies showing an overall lower prevalence and delayed incidence of cardiovascular atherosclerotic disease in female patients (20, 21). Considering the higher rates of beneficial outcome in female patients, our data provide a reason to consider VA-ECMO as a therapy option for pulmonary embolism deteriorating to CA, supporting the present findings suggesting that this might be a feasible therapeutic strategy (22).

The reason for the higher rates of bleeding complications in female patients remains unclear. A potential explanation could be the higher rates of pulmonary embolism in female patients and therefore possible administration of thrombolytic therapy prior to eCPR initiation, accounting for higher rates of periprocedural bleeding due to the invasive nature of VA-ECMO implantation. Furthermore, these findings might derive from gender-specific anatomical differences. Female patients are known to have significantly smaller iliofemoral arterial diameters potentially complicating vascular access (23). This might also justify the gender-specific differences in the impact of BMI on adverse outcomes. Regarding coronary interventions, data suggest an increased risk for periinterventional all-cause mortality for underweight to normoweight female patients (24). Our data suggest that these findings might be transferable to the context of eCPR.

Specifically shockable initial rhythm, witnessed collapse, and performance of bystander CPR are the known predictors for a favourable outcome and are therefore found in the current inclusion criteria for eCPR (25). The results from our multivariable analysis support the impact of these factors.

### 4.1 Limitations

The present analysis is based on retrospective data from a single centre and thus might not be applicable to the general population. Accounting for 21% (*n* = 69) of the study population, the percentage of female patients in our study was higher compared with that of present RCTs, but it was still below the percentage of female patients suffering OHCA described in the current registry data (34%) (26). Furthermore, our results are limited by the long study timespan, portraying an inherent learning curve, not only regarding the management of eCPR patients, but also the identification and selection of patients susceptible to this therapy. We report comparatively high rates of non-shockable initial rhythm impeding comparison with current RCTs. While the decision to implement eCPR remains an individualised decision, the trend has shifted to limit this therapy to patients presenting with shockable initial rhythm. The apparent selection bias, as to whom is deemed suitable for this highly invasive and resource-demanding therapeutic option, has to be reported as a major constraint, not only in the present study, but in all investigations in the field of eCPR. In addition, the earlier-described limited availability of mCPR devices during the study period might have considerably affected resuscitation durations and therefore potentially outcome parameters.

## 5 Conclusion

Gender differences are relevant for favourable outcomes in patients undergoing eCPR for OHCA. Along with the underrepresentation of female gender in the present studies, this calls for further in-depth investigation and potentially consecutive individualisation of therapy.

## Data Availability

The raw data supporting the conclusions of this article will be made available by the authors, without undue reservation.
